# Draft genome sequence data of *Paenbacillus curdlanolyticus* B-6 possessing a unique xylanolytic-cellulolytic multienzyme system

**DOI:** 10.1016/j.dib.2020.106213

**Published:** 2020-08-22

**Authors:** Sirilak Baramee, Ayaka Uke, Chakrit Tachaapaikoon, Rattiya Waeonukul, Patthra Pason, Khanok Ratanakhanokchai, Akihiko Kosugi

**Affiliations:** a Biological Resources and Post-Harvest Division, Japan International Research Center for Agricultural Sciences (JIRCAS), 1-1 Ohwashi, Tsukuba, Ibaraki 305-8686, Japan; b Pilot Plant Development and Training Institute (PDTI), King Mongkut's University of Technology Thonburi (KMUTT), Bangkok 10150, Thailand; c Enzyme Technology Laboratory, School of Bioresources and Technology, King Mongkut's University of Technology Thonburi (KMUTT), Bangkok 10150, Thailand

**Keywords:** *Paenibacillus curdlanolyticus* B-6, Draft genome, Xylanolytic enzyme, Cellulolytic enzyme, Multienzyme complex

## Abstract

*Paenibacillus curdlanolyticus* B-6 is a facultative anaerobic bacterium that efficiently produces a lignocellulolytic multienzyme complex. The whole genome of *P. curdlanolyticus* B-6 was sequenced on an Ion GeneStudio S5 system, which yielded 74 contigs with a total size of 4,875,097 bp, 4,473 protein-coding sequences, and a G+C content of 49.7%. The genome data have been deposited in DDBJ/ENA/GenBank under accession numbers BLWM01000001–BLWM01000074. Analyses of average nucleotide identities and phylogenetic relationships of 16S rRNA sequences of *Paenibacillus* species revealed that strain B-6 is most closely related to *Paenibacillus xylaniclasticus* TW1. *P. curdlanolyticus* B-6 should thus be reclassified as a strain of *P. xylaniclasticus*.

**Specifications Table**

**Table:** 

Subject	Microbiology
Specific subject area	Bacteriology, Genomics
Type of data	Table, Figures
How data were acquired	Whole-genome sequencing using Ion GeneStudio S5 System
Data format	Raw and Analyzed
Parameters for data collection	Genomic DNA was extracted from pure culture of *P. curdlanolyticus* B-6. The genome of strain B-6 was sequenced by using Ion GeneStudio S5 System, *de novo* assembled using CLC Genomic Workbench 20.0.1, and annotated using DDBJ Fast Annotation and Submission Tool (DFAST).
Description of data collection	Genomic DNA extracted from *P. curdlanolyticus* B-6, following whole-genome sequencing, assembly, and annotation
Data source location	Japan International Research Center for Agricultural Sciences (JIRCAS) Tsukuba, Ibaraki, Japan
Data accessibility	Repository name: DDBJ/ENA/GenBank Data identification number: BLWM01000000. The version described in this paper is BLWM01000000.1 Direct URL to data: https://www.ncbi.nlm.nih.gov/nuccore/BLWM00000000.1 The BioProject ID in GenBank is PRJDB9861 (https://www.ncbi.nlm.nih.gov/bioproject/PRJDB9861) The BioSample ID in GenBank is SAMD00228050 (https://www.ncbi.nlm.nih.gov/biosample/?term=SAMD00228050)
Related research article	P. Pason, K-L. Kyu, K. Ratanakhanokchai, *Paenibacillus curdlanolyticus* strain B-6 xylanolytic-cellulolytic enzyme system that degrades insoluble polysaccharides, Appl. Environ. Microbiol. 72 (2006) 2483–2490. 10.1128/AEM.72.4.2483–2490.2006

## Value of the Data

• *Paenibacillus curdlanolyticus* B-6 produces a large extracellular complex enzyme, which is unusual in cellulolytic-xylanolytic *Paenibacillus* species.
• Genome data of strain B-6 will be useful for further functional genomics and enzyme engineering research.
• The draft genome sequence of strain B-6 can aid understanding of the polysaccharide degradation mechanism of this bacterium and may be useful as a reference sequence for *Paenibacillus* species classification.

## 1 Data Description

Bacterial enzyme systems for lignocellulose degradation can be generally regarded as non-complexed or complexed enzymes that are normally produced by aerobic and anaerobic bacteria, respectively. In terms of hydrolysis efficiency, the complexed enzymes offer greater potential for the degradation of lignocellulose compared with non-complexed ones. The production of enzymes by anaerobic culture is very costly, however, mainly because of the high price of medium, slow rate of growth, and low enzyme yield [1].

The mesophilic facultatively anaerobic bacterium *Paenibacillus curdlanolyticus* strain B-6, isolated from an anaerobic digester fed with pineapple wastes [2], was originally classified according to the results of a 16S rRNA gene analysis by Pason et al. [3]. Strain B-6 is a true lignocellulolytic microorganism, as it can use xylan, microcrystalline cellulose, and lignocellulosic biomass as sole carbon sources [3]. Strain B-6 was found to produce complexed enzymes under aerobic conditions [4, 5], a rarely reported phenomenon [6,7,8,9]. In recent years, the characteristics and function of the lignocellulolytic enzyme system of this bacterium have been the subject of considerable research. We found that the complex enzyme produced by strain B-6 is critical for improving lignocellulosic biomass degradation; however, the mechanisms of lignocellulose degradation and utilization are still unclear. A similar bacterial example, *Paenibacillus xylaniclasticus* strain TW1 [10] isolated from sludge in an anaerobic digester, is known to have a xylan degradation system, as in strain B-6. Because of differences in several phenotypic characters, such as growth temperature and acid formation [10], we have not previously analyzed the taxonomic relationship of strains B-6 and TW1. An understanding of the genetic relationship of the two strains and differences in their xylan degradation systems was thus needed.

In this work, we determined the draft genome sequence of strain B-6 to obtain further information on lignocellulose utilization systems in the genus *Paenibacillus*. Features of the genome are shown in Table 1. DNA sequencing, performed using the Ion GeneStudio S5 System, generated 45,085,168 reads. The genome was assembled de novo using CLC Genomic Workbench 20.0.1 (CLC Bio, Qiagen, Valencia, CA), which resulted in 74 contigs with an N50 of 237,553 bp and a maximum size of 430,139 bp. The genome of *P. curdlanolyticus* strain B-6 comprised 4,875,097 bp and had a *G* + *C* content of 49.7%, which is nearly identical to that of *P. xylaniclasticus* (4,924,585 bp, with a *G* + *C* content of 49.6%). Genome annotation was performed with the DDBJ Fast Annotation and Submission Tool (DFAST). *Paenibacillus curdlanolyticus* strain B-6 was found to have 4,473 protein-coding sequences (CDSs), 4 rRNA genes, and 94 tRNA genes.

**Table 1:** Features of the *Paenibacillus curdlanolyticus* B-6 genome.

Feature	Description
Number of reads used in the assembly	45,085,168
Mean read length	195 bp
Genome size	4,875,097 bp
Number of contigs	74
*G* + *C* content (%)	49.7
N50 contig length	237,553 bp
Mean contig length	65,880 bp
Number of CDSs	4,473
Number of rRNAs	4
Number of tRNAs	94
Number of CRISPRs	5
Genome coverage depth	1,718-fold

**Fig. 1:**
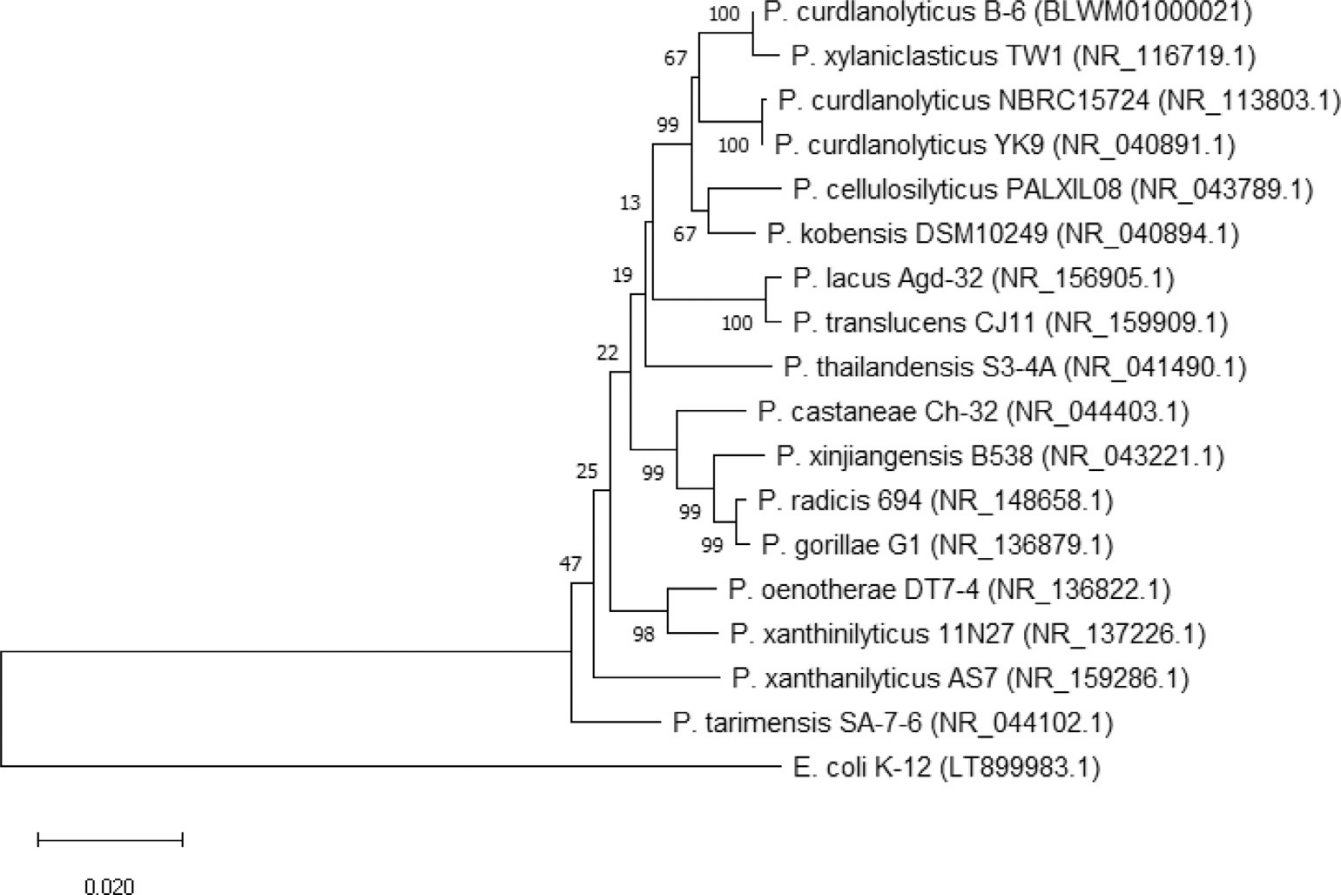
A 16S rRNA-based phylogenetic tree of *Paenibacillus curdlanolyticus* B-6 and related members of the genus *Paenibacillus. Escherichia coli* K-12 was used as an outgroup. The phylogenetic tree was constructed using the neighbor-joining method with 1,000 bootstrap replicates. The bar represents 0.02 substitutions per nucleotide position.

**Fig. 2:**
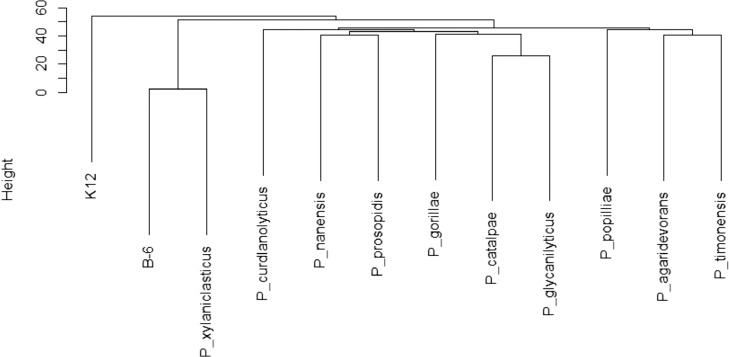
Dendrogram of average nucleotide identity (ANI) values. The ANI value of each strain was calculated, and a dendrogram was constructed using the unweighted pair group method with arithmetic means. *Escherichia coli* K-12 (NZ_CP014272) was used as an outgroup. Strains of *Paenibacillus* were as follows: *Paenibacillus agaridevorans* (NZ_BDQX01000000), *P. catalpae* (NZ_FOMT01000000), *P. curdlanolyticus* (NZ_AEDD01000000), *P. glycanilyticus* (NZ_BILY01000000), *P. gorillae* (NZ_CBVJ010000000), *P. nanensis* (NZ_QXQA01000000), *P. popilliae* (NZ_BALG01000000), *P. prosopidis* (NZ_QPJD01000000), *P. timonensis* (NZ_WNZY01000000), and *P. xylaniclasticus* (NZ_BIML01000000).

Phylogenetic analysis of 16S rRNA sequences of strain B-6 and 16 related strains revealed that strain B-6 is very closely related to *P. xylaniclasticus* TW1 (99.51% similarity), *P. curdlanolyticus* NBRC15724 (97.74% similarity), and *P. curdlanolyticus* YK9 (97.67% similarity) (Fig. 1 and Suppl. Table 1). Moreover, an assay of the average nucleotide identity (ANI) [11] of 11 strains belonging in 10 *Paenibacillus* species, including strain B-6, and one outgroup strain, *Escherichia coli* K-12 (LT899983), showed that strain B-6 is more closely related to *P. xylaniclasticus* TW1 than to *P. curdlanolyticus* YK9 (Fig. 2, Suppl. Table 2 and 3).

Although analysis of the genome data of strain B-6 demonstrated its high similarity to strain TW1, a previous investigation of enzyme component patterns of both strains clearly indicated they have different xylanase profiles [3, 10]. In addition, BLAST searching with the B-6 draft sequence as the query failed to uncover two characteristic xylanases of strain B-6, namely, Xyn10D [12] and Xyn10E [13], in the *P. xylaniclasticus* TW1 genome. We therefore believe that taxonomic analysis of strains B-6 and TW1 is necessary.

## 2 Experimental design, materials and methods

### 2.1 Genomic DNA extraction and sequencing

Genomic DNA of *P. curdlanolyticus* B-6 was obtained by phenol/chloroform extraction from cells grown under aerobic conditions at 37 °C. Fragmentation of DNA was performed with a Bioruptor sonicator (BMBio, Japan), which generated fragments with an average length of 500 bp. Approximately 400- to 600-bp fragments were size-selected by electrophoresis on E-Gel SizeSelect II agarose gels (Invitrogen, Thermo Fisher Scientific) before library preparation. The DNA library was prepared using an Ion Plus Fragment Library kit (Thermo Fisher Scientific) according to the manufacturer's protocol. The genomic DNA of *P. curdlanolyticus* B-6 was sequenced using an Ion GeneStudio S5 System.

### 2.2 Phylogenetic species identification

The 16S rRNA sequence of strain B-6 was analyzed using the BLAST search engine and manually aligned with sequences in the GenBank database using the Multiple Sequence Alignment option in CLUSTAL W (https://www.genome.jp/tools-bin/clustalw). Phylogenetic trees were constructed by the neighbor-joining method using MEGA version 10.1.8 software [14]. Tree topologies and distances were estimated by performing a bootstrap analysis with 1,000 re-samplings.

### 2.3 Genome assembly and annotation

After removal of low-quality reads, *de novo* genome assembly was performed using CLC Genomic Workbench version 20.0.1. The genome was annotated using DFAST (https://dfast.nig.ac.jp/). An additional analysis was performed using the carbohydrate-active enzymes (CAZy) database (http://www.cazy.org/).

### 2.4 Genomic ANI

Pairwise ANI values of whole genome sequences of *Paenibacillus* strains were calculated using GENETYX NGS version 4.1.1. The matrix generated from ANI values among *Paenibacillus* strains was converted to a genetic dendrogram using algorithms such as the unweighted pair group method with arithmetic means and the single-linkage clustering method in the R statistical program.

## Declaration of Competing Interest

The authors declare that they have no known competing financial interests or personal relationships which have, or could be perceived to have, influenced the work reported in this article.

## Appendix Supplementary materials

Image, application 1

Image, application 2

Image, application 3

Image, application 4

Image, application 5
