# Supplementary material for: Draft genome sequence data of Paenbacillus curdlanolyticus B-6 possessing a unique xylanolytic-cellulolytic multienzyme system
Source: Data Brief. 2020 Aug 22;32:106213. doi: 10.1016/j.dib.2020.106213 (PMC7476223; doi:10.1016/j.dib.2020.106213)
Supplement: Supplementary file 3 [file mmc3.docx]

Supplementary Table 1. List of 16S rRNA-based phylogenetic tree of *Paenibacillus curdlanolyticus* B-6 and related members of the genus *Paenibacillus*.

| Organisms | Strain | Accession no. |
| --- | --- | --- |
| *Paenibacillus curdlanolyticus* | B-6 | BLWM01000021 |
| *Paenibacillus curdlanolyticus* | NBRC 15724 | NR_113803.1 |
| *Paenibacillus curdlanolyticus* | YK9 | NR_040891.1 |
| *Paenibacillus xylaniclasticus* | TW1 | NR_116719.1 |
| *Paenibacillus cellulosilyticus* | PALXIL08 | NR_043789.1 |
| *Paenibacillus kobensis* | DSM 10249 | NR_040894.1 |
| *Paenibacillus radicis* | 694 | NR_148658.1 |
| *Paenibacillus lacus* | Agd-32 | NR_156905.1 |
| *Paenibacillus gorillae* | G1 | NR_136879.1 |
| *Paenibacillus translucens* | CJ11 | NR_159909.1 |
| *Paenibacillus oenotherae* | DT7-4 | NR_136822.1 |
| *Paenibacillus thailandensis* | S3-4A | NR_041490.1 |
| *Paenibacillus castaneae* | Ch-32 | NR_044403.1 |
| *Paenibacillus xanthinilyticus* | 11N27 | NR_137226.1 |
| *Paenibacillus xinjiangensis* | B538 | NR_043221.1 |
| *Paenibacillus xanthanilyticus* | AS7 | NR_159286.1 |
| *Paenibacillus tarimensis* | SA-7-6 | NR_044102.1 |
| *Escherichia coli* | K-12 | LT899983.1 |

Supplementary Table 2. *Paenibacillus* isolates used in dendrogram of average nucleotide identity (ANI) values.

| Organisms | Accession no. |
| --- | --- |
| *Paenibacillus agaridevorans* | NZ_BDQX01000000 |
| *Paenibacillus catalpae* | NZ_FOMT01000000 |
| *Paenibacillus curdlanolyticus* | NZ_AEDD01000000 |
| *Paenibacillus glycanilyticus* | NZ_BILY01000000 |
| *Paenibacillus gorillae* | NZ_CBVJ010000000 |
| *Paenibacillus nanensis* | NZ_QXQA01000000 |
| *Paenibacillus popilliae* | NZ_BALG01000000 |
| *Paenibacillus prosopidis* | NZ_QPJD01000000 |
| *Paenibacillus timonensis* | NZ_WNZY01000000 |
| *Paenibacillus xylaniclasticus* | NZ_BIML01000000 |
| *Escherichia coli* | NZ_CP014272 |

Supplementary Table 3. Pairwise ANI values among *Paenibacillus curdlanolyticus* B-6, other *Paenibacillus* strains, and the outgroup strain *Escherichia coli* K-12.

|  | Strain B-6 | *P. agaridevorans* | *P. catalpae* | *P. curdlanolyticus* | *P. glycanilyticus* | *P. gorillae* | *P. nanensis* | *P. popilliae* | *P. prosopidis* | *P. timonensis* | *P. xylaniclasticus* | *E. coli* K-12 |
| --- | --- | --- | --- | --- | --- | --- | --- | --- | --- | --- | --- | --- |
| Strain B-6 | 100.0 |  |  |  |  |  |  |  |  |  |  |  |
| *P. agaridevorans* | 69.9 | 100.0 |  |  |  |  |  |  |  |  |  |  |
| *P. catalpae* | 70.0 | 69.3 | 100.0 |  |  |  |  |  |  |  |  |  |
| *P. curdlanolyticus* | 73.9 | 68.4 | 69.6 | 100.0 |  |  |  |  |  |  |  |  |
| *P. glycanilyticus* | 69.9 | 68.5 | 81.3 | 69.7 | 100.0 |  |  |  |  |  |  |  |
| *P. gorillae* | 70.1 | 69.7 | 71.9 | 69.9 | 71.8 | 100.0 |  |  |  |  |  |  |
| *P. nanensis* | 69.7 | 70.8 | 69.8 | 69.5 | 70.1 | 70.2 | 100.0 |  |  |  |  |  |
| *P. popilliae* | 68.7 | 68.8 | 68.2 | 68.8 | 68.6 | 68.8 | 69.2 | 100.0 |  |  |  |  |
| *P. prosopidis* | 69.4 | 70.8 | 70.7 | 69.7 | 70.6 | 71.1 | 71.4 | 68.1 | 100.0 |  |  |  |
| *P. timonensis* | 68.0 | 67.9 | 67.5 | 68.2 | 68.0 | 68.0 | 68.2 | 68.7 | 67.7 | 100.0 |  |  |
| *P. xylaniclasticus* | 98.0 | 69.7 | 70.0 | 73.5 | 70.2 | 70.1 | 70.0 | 68.3 | 69.7 | 68.1 | 100.0 |  |
| *E. coli* K-12 | 64.8 | 64.3 | 63.6 | 66.0 | 63.2 | 65.0 | 63.4 | 64.9 | 64.3 | 63.9 | 65.4 | 100.0 |
