# Supplementary material for: Draft genome sequence data of Paenbacillus curdlanolyticus B-6 possessing a unique xylanolytic-cellulolytic multienzyme system
Source: Data Brief. 2020 Aug 22;32:106213. doi: 10.1016/j.dib.2020.106213 (PMC7476223; doi:10.1016/j.dib.2020.106213)
Supplement: Supplementary file 4 [file mmc4.docx]

>Paenibacillus curdlanolyticus B-6 (BLWM01000021)

GCAAGTCGAGCGGACTTGATGGAGTGCTTGCACTCCTGAGAGTTAGCGGCGGACGGGTGAGTAACACGTAGGCAACCTGCCCGTAAGACTGGGATAACATTCGGAAACGAATGCTAATACCAGATACGCGATTTCCTCGCATGGGGGAGTCGGGAAAGATGGAGCAATCTATCACTTACGGATGGGCCTGCGGCGCATTAGCTAGTTGGTGGGGTAACGGCCTACCAAGGCGACGATGCGTAGCCGACCTGAGAGGGTGATCGGCCACACTGGGACTGAGACACGGCCCAGACTCCTACGGGAGGCAGCAGTAGGGAATCTTCCGCAATGGACGCAAGTCTGACGGAGCAACGCCGCGTGAGTGAAGAAGGCTTTCGGGTCGTAAAGCTCTGTTGCCAGGGAAGAACGCTTGGGAGAGTAACTGCTCCCAAGGTGACGGTACCTGAGAAGAAAGCCCCGGCTAACTACGTGCCAGCAGCCGCGGTAATACGTAGGGGGCAAGCGTTGTCCGGAATTATTGGGCGTAAAGCGCGCGCAGGCGGCTTTGTAAGTCTGTCGTTTAAGTTCGGGGCTCAACCCCGTATCGCGATGGAAACTGCAAGGCTTGAGTGCAGAAGAGGAAAGTGGAATTCCACGTGTAGCGGTGAAATGCGTAGAGATGTGGAGGAACACCAGTGGCGAAGGCGACTTTCTGGGCTGTAACTGACGCTGAGGCGCGAAAGCGTGGGGAGCAAACAGGATTAGATACCCTGGTAGTCCACGCCGTAAACGATGAATGCTAGGTGTTAGGGGTTTCAATACCCTTGGTGCCGAAGTTAACACATTAAGCATTCCGCCTGGGGAGTACGCTCGCAAGAGTGAAACTCAAAGGAATTGACGGGGACCCGCACAAGCAGTGGAGTATGTGGTTTAATTCGAAGCAACGCGAAGAACCTTACCAGGTCTTGACATCCCTCTGACCGCTCTAGAGATAGGGCTTCCCTTCGGGGCAGAGGAGACAGGTGGTGCATGGTTGTCGTCAGCTCGTGTCGTGAGATGTTGGGTTAAGTCCCGCAACGAGCGCAACCCTTGATTTTAGTTGCCAGCATTTCGGATGGGCACTCTAGAATGACTGCCGGTGACAAACCGGAGGAAGGCGGGGATGACGTCAAATCATCATGCCCCTTATGACCTGGGCTACACACGTACTACAATGGCCGGTACAACGGGCAGCGAAGGAGCGATCTGGAGCGAATCCTATAAAGCCGGTCTCAGTTCGGATTGCAGGCTGCAACTCGCCTGCATGAAGTCGGAATTGCTAGTAATCGCGGATCAGCATGCCGCGGTGAATACGTTCCCGGGTCTTGTACACACCGCCCGTCACACCACGAGAGTTTACAACACCCGAAGCCGGTGGGGTAACCGCAAGGAGCCAGC

>Paenibacillus xylaniclasticus TW1 (NR_116719.1)

AGAGTTTGATCCTGGCTCAGGACGAACGCTGGCGGCGTGCCTAATACATGCAAGTCGAGCGGACTTGATGGAGTGCTTGCACTCCTGAGAGTTAGCGGCGGACGGGTGAGTAACGCGTAGGCAACCTGCCCGTAAGACTGGGATAACATTCGGAAACGAATGCTAATACCAGATACGCGATTTCCTCGCATGGGGGAGTCGGGAAAGATGGAGCAATCTATCACTTACGGATGGGCCTGCGGCGCATTAGCTAGTTGGTGGGGTAACGGCCTACCAAGGCGACGATGCGTAGCCGACCTGAGAGGGTGATCGGCCACACTGGGACTGAGACACGGCCCAGACTCCTACGGGAGGCAGCAGTAGGGAATCTTCCGCAATGGACGCAAGTCTGACGGAGCAACGCCGCGTGAGTGAAGAAGGCTTTCGGGTCGTAAAGCTCTGTTGCCAGGGGAGAACGCTTGGGAGAGTAACTGCTCCCAAGGTGACGGTACCTGAGAAGAAAGCCCCGGCTAACTACGTGCCAGCAGCCGCGGTAATACGTAGGGGGCAAGCGTTGTCCGGAATTATTGGGCGTAAAGCGCGCGCAGGCGGCTTTGTGAGTCTGTCGTTTAAGTTCGGGGCTCAACCCCGTATCGCGATGGAAACTGCAAGGCTTGAGTGCAGAAGAGGAAAGTGGAATTCCACGTGTAGCGGTGAAATGCGTAGAGATGTGGAGGAACACCAGTGGCGAAGGCGACTTTCTGGGCTGTAACTGACGCTGAGGCGCGAAAGCGTGGGGAGCAAACAGGATTAGATACCCTGGTAGTCCACGCCGTAAACGATGAGTGCTAGGTGTTAGGGGTTTCAATACCCTTGGTGCCGAAGTTAACACATTAAGCATTCCGCCTGGGGAGTACGCTCGCAAGAGTGAAACTCAAAGGAATTGACGGGGACCCGCACAAGCAGTGGAGTATGTGGTTTAATTCGAAGCAACGCGAAGAACCTTACCAGGTCTTGACATCCCTCTGACCGCTCTAGAGATAGGGCTTCCCTTCGGGGCAGAGGAGACAGGTGGTGCATGGTTGTCGTCAGCTCGTGTCGTGAGATGTTGGGTTAAGTCCCGCAACGAGCGCAACCCTTGATTTTAGTTGCCAGCATTTCGGATGGGCACTCTAGAATGACTGCCGGTGACAAACCGGAGGAAGGCGGGGATGACGTCAAATCATCATGCCCCTTATGACCTGGGCTACACACGTACTACAATGGCCGGTACAACGGGCAGCGAAGGAGCGATCTGGAGCGAATCCTATAAAGCCAGTCTCAGTTCGGATTGCAGGCTGCAACCCGCCTGCATGAAGTCGGAATTGCTAGTAATCGCGGATCAGCATGCCGCGGTGAATACGTTCCCGGGTCTTGTACACACCGCCCGTCACACCACGAGAGTTTACAACACCCGAAACCGGTGGGGTAACCGCAAGGAGCCAGCCGTCGAAGGTGGGGTAGATGATTGGGGTGAAGTCGTAACAAGGTAACC

>Paenibacillus curdlanolyticus NBRC 15724 (NR_113803.1)

GACGAACGCTGGCGGCGTGCCTAATACATGCAAGTCGAGCGGACTTGATGGAGTGCTTGCACTCCTGATAGTTAGCGGCGGACGGGTGAGTAACACGTAGGCAACCTGCCCGTAAGACTGGGATAACATTCGGAAACGAATGCTAATACCGGATACGCGATTTTCTCGCATGAGNGAATCGGGAAAGAAGGAGCAATCTTTCACTTACGGATGGGCCTGCGGCGCATTAGCTAGTTGGTGGGGTAATGGCTCACCAAGGCGACGATGCGTAGCCGACCTGAGAGGGTGATCGGCCACACTGGGACTGAGACACGGCCCAGACTCCTACGGGAGGCAGCAGTAGGGAATCTTCCGCAATGGACGAAAGTCTGACGGAGCAACGCCGCGTGAGTGAAGAAGGCTTTCGGGTCGTAAAGCTCTGTTGCCAGGGAAGAACACTTGAGAGAGTAACTGCTCTTGAGTTGACGGTACCTGAGAAGAAAGCCCCGGCTAACTACGTGCCAGCAGCCGCGGTAATACGTAGGGGGCAAGCGTTGTCCGGAATTATTGGGCGTAAAGCGCGCGCAGGCGGCTTTGTAAGTCTGTCGTTTAAGTTCGGGGCTCAACCCCGTGTCGCGATGGAAACTGCAAGGCTTGAGTACAGAAGAGGAAAGTGGAATTCCACGTGTAGCGGTGAAATGCGTAGAGATGTGGAGGAACACCAGTGGCGAAGGCGACTTTCTGGGCTGTAACTGACGCTGAGGCGCGAAAGCGTGGGGAGCAAACAGGATTAGATACCCTGGTAGTCCACGCCGTAAACGATGAATGCTAGGTGTTAGGGGTTTCAATACCCTTGGTGCCGAAGTTAACACATTAAGCATTCCGCCTGGGGAGTACGCTCGCAAGAGTGAAACTCAAAGGAATTGACGGGGACCCGCACAAGCAGTGGAGTATGTGGTTTAATTCGAAGCAACGCGAAGAACCTTACCAGGTCTTGACATCCCTCTGACCGTCGCAGAGATGTGACTTCCCTTCGGGGCAGAGGAGACAGGTGGTGCATGGTTGTCGTCAGCTCGTGTCGTGAGATGTTGGGTTAAGTCCCGCAACGAGCGCAACCCTTGATTTTAGTTGCCAGCACTTTAAGGTGGGCACTCTAGAATGACTGCCGGTGACAAACCGGAGGAAGGCGGGGATGACGTCAAATCATCATGCCCCTTATGACCTGGGCTACACACGTACTACAATGGCCGGTACAACGGGCTGCGAAAGAGCGATCTGGAGCGAATCCTATAAAGCCGGTCTCAGTTCGGATTGCAGGCTGCAACTCGCCTGCATGAAGTCGGAATTGCTAGTAATCGCGGATCAGCATGCCGCGGTGAATACGTTCCCGGGTCTTGTACACACCGCCCGTCACACCACGAGAGTTTACAACACCCGAAGCCGGTGGGGTAACCGCAAGGAGCCAGCCGNCAAAGGTGGGGNAAATGATTGGGGTGAAG

>Paenibacillus curdlanolyticus YK9 (NR_040891.1)

GATCATGGCTCAGGACGAACGCTGGCGGCGTGCCTAATACATGCAAGTCGAGCGGACTTGATGGAGTGCTTGCACTCCTGANNGTTAGCGGCGGACGGGTGAGTAACACGTAGGCAACCTGCCCGTAAGACTGGGATAACATTCGGAAACGAATGCTAATACCGGATACGCGATTTTCTCGCATGAGAGAATCGGGAAAGAAGGAGCAATCTTTCACTTACGGATGGGCCTGCGGCGCATTAGCTAGTTGGTGGGGTAATGGCTCACCAAGGCGACGATGCGTAGCCGACCTGAGAGGGTGATCGGCCACACTGGGACTGAGACACGGCCCAGACTCCTACGGGAGGCAGCAGTAGGGAATCTTCCGCAATGGACGAAAGTCTGACGGAGCAACGCCGCGTGAGTGAAGAAGGCTTTCGGGTCGTAAAGCTCTGTTGCCAGGGAAGAACACTTGAGAGAGTAACTGCTCTTGAGTTGACGGTACCTGAGAAGAAAGCCCCGGCTAACTACGTGCCAGCAGCCGCGGTAATACGTAGGGGGCAAGCGTTGTCCGGAATTATTGGGCGTAAAGCGCGCGCAGGCGGCTTTGTAAGTCTGTCGTTTAAGTTCGGGGCTCAACCCCGTGTCGCGATGGAAACTGCAAGGCTTGAGTACAGAAGAGGAAAGTGGAATTCCACGTGTAGCGGTGAAATGCGTAGAGATGTGGAGGAACACCAGTGGCGAAGGCGACTTTCTGGGCTGTAACTGACGCTGAGGCGCGAAAGCGTGGGGAGCAAACAGGATTAGATACCCTGGTAGTCCACGCCGTAAACGATGAATGCTAGGTGTTAGGGGTTTCAATACCCTTGGTGCCGAAGTTAACACATTAAGCATTCCGCCTGGGGAGTACGCTCGCAAGAGTGAAACTCAAAGGAATTGACGGGGACCCGCACAAGCAGTGGAGTATGTGGTTTAATTCGAAGCAACGCGAAGAACCTTACCAGGTCTTGACATCCCTCTGACCGTCGCAGAGATGTGACTTCCCTTCGGGGCAGAGGAGACAGGTGGTGCATGGTTGTCGTCAGCTCGTGTCGTGAGATGTTGGGTTAAGTCCCGCAACGAGCGCAACCCTTGATTTTAGTTGCCAGCACTTTANGGTGGGCACTCTAGAATGACTGCCGGTGACAAACCGGAGGAAGGCGGGGATGACGTCAAATCATCATGCCCCTTATGACCTGGGCTACACACGTACTACAATGGCCGGTACAACGGGCTGCGAAAGAGCGATCTGGAGCGAATCCTATAAAGCCGGTCTCAGTTCGGATTGCAGGCTGCAACTCGCCTGCATGAAGTCGGAATTGCTAGTAATCGCGGATCAGCATGCCGCGGTGAATACGTTCCCGGGTCTTGTACACACCGCCCGTCACACCACGAGAGTTTACAACACCCGAAGCCGGTGGGGTAACCGCAAGGAGCCAGCCGTCGAAGGTGGGGTAGATGATTGGGGTGAAGTCGTAACAAGGTAGCCGTATCGGAAGGTGCGGCTGGATCA

>Paenibacillus cellulosilyticus PALXIL08 (NR_043789.1)

TAGAGTTTGATCCTGGCTCAGGACGAACGCTGGCGGCGTGCCTAATACATGCAAGTCGAGCGGAGTTGATGAGGTGCTTGCACCTCTGATGCTTAGCGGCGGACGGGTGAGTAACACGTAGGCAACCTGCCTGCAGGACTGGGATAACATTCGGAAACGAATGCTAATACCGGATAATCGATTTCCTCGCATGGGGAGATCGGGAAAGACGGAGCAATCTGTCACCTGTGGATGGGCCTGCGGCGCATTAGCTAGTTGGTGGGGTAACGGCCTACCAAGGCGACGATGCGTAGCCGACCTGAGAGGGTGATCGGCCACACTGGGACTGAGACACGGCCCAGACTCCTACGGGAGGCAGCAGTAGGGAATCTTCCGCAATGGACGAAAGTCTGACGGAGCAACGCCGCGTGAGTGATGAAGGCTTTCGGGTCGTAAAGCTCTGTTGCCAGGGAAGAACACTTGGGAGAGTAACTGCTCTTAAGGTGACGGTACCTGAGAAGAAAGCCCCGGCTAACTACGTGCCAGCAGCCGCGGTAATACGTAGGGGGCAAGCGTTGTCCGGAATTATTGGGCGTAAAGCGCGCGCAGGCGGCTTTGTAAGTCTGTCGTTTAAGTTCGGGGCTCAACCCCGTATCGCGATGGAAACTGCAAGGCTTGAGTGCAGAAGAGGAAAGTGGAATTCCACGTGTAGCGGTGAAATGCGTAGAGATGTGGAGGAACACCAGTGGCGAAGGCGACTTTCTGGGCTGTAACTGACGCTGAGGCGCGAAAGCGTGGGGAGCAAACAGGATTAGATACCCTGGTAGTCCACGCCGTAAACGATGAATGCTAGGTGTTAGGGGTTTCAATACCCTTGGTGCCGAAGTTAACACATTAAGCATTCCGCCTGGGGAGTACGCTCGCAAGAGTGAAACTCAAAGGAATTGACGGGGACCCGCACAAGCAGTGGAGTATGTGGTTTAATTCGAAGCAACGCGAAGAACCTTACCAGGTCTTGACATCCCTCTGACCGGTCTGGAGACAGGCCTTCCCTTCGGGACAGAGGAGACAGGTGGTGCATGGTTGTCGTCAGCTCGTGTCGTGAGATGTTGGGTTAAGTCCCGCAACGAGCGCAACCCTTGATTTTAGTTGCCAGCACTTCGGATGGGCACTCTAGAATGACTGCCGGTGACAAACCGGAGGAAGGCGGGGATGACGTCAAATCATCATGCCCCTTATGACCTGGGCTACACACGTACTACAATGGCCGGTACAACGGGCTGCGAAGGAGCGATCCGGAGCGAATCCTATAAAGCCGGTCTCAGTTCGGATTGGAGGCTGCAACTCGCCTCCATGAAGTCGGAATTGCTAGTAATCGCGGATCAGCATGCCGCGGTGAATACGTTCCCGGGTCTTGTACACACCGCCCGTCACACCACGAGAGTTTACAACACCCGAAGCCGGTGGGGTAACCGCAAGGAGCCAGCCGTCGAAGGTGGGGTAGATGATTGGGGTGAAGTCGTAACAAGGTAGCCGTATCGGAAGGTGTGGNTGGATCACCTCCTTA

>Paenibacillus kobensis DSM 10249 (NR_040894.1)

CGAACGCTGGCGGCGTGCCTAATACATGCAAGTCGAGCGGATCTGAAGGAGTGCTTGCACTCCTGATGGTTAGCGGCGGACGGGTGAGTAACACGTAGGTAACCTGCCTACAAGACNGGGATAACATTCGGAAACGAATGCTAATACCGGATACGCGATTTCCTCGCATGGGGAGATCGGGAAAGACGGAGCAATCTGTCACTTGTAGATGGACCTGCGGCGCATTAGCTAGTTGGTGGGGTAACGGCTCACCAAGGCGACGATGCGTAGCCGACCTGAGAGGGTGATCGGCCACACTGGGACTGAGACACGGCCCAGACTCCTACGGGAGGCAGCAGTAGGGAATCTTCCGCAATGGACGAAAGTCTGACGGAGCAACGCCGCGTGAGTGATGAAGGCTTTCGGGTCGTAAAGCTCTGTTGCCAGGGAAGAACGCTTGGGAGAGTAACTGCTCTCAAGGTGACGGTACCTGAGAAGAAAGCCCCGGCTAACTACGTGCCAGCAGCCGCGGTAATACGTAGGGGGCAAGCGTTGTCCGGAATTATTGGGCGTAAAGCGCGCGCAGGCGGCTTTGTAAGTCTGTCGTTTAAGTTCGGGGCTCAACCCCGTATCGCGATGGAAACTGCAAGGCTTGAGTACAGAAGAGGAAAGTGGAATTCCACGTGTAGCGGTGAAATGCGTAGAGATGTGGAGGAACACCAGTGGCGAAGGCGACTTTCTGGGCTGTAACTGACGCTGAGGCGCGAAAGCGTGGGGAGCAAACAGGATTAGATACCCTGGTAGTCCACGCCGTAAACGATGAATGCTAGGTGTTAGGGGTTTCAATACCCTTGGTGCCGAAGTTAACACATTAAGCATTCCGCCTGGGGAGTACGCTCGCAAGAGTGAAACTCAAAGGAATTGACGGGGACCCGCACAAGCAGTGGAGTATGTGGTTTAATTCGAAGCAACGCGAAGAACCTTACCAGGTCTTGACATCCCTNTGACCGTCCTAGAGATAGGGCTTCCCTTCGGGGCAGAGGAGACAGGTGGTGCATGGTTGTCGTCAGCTCGTGTCGTGAGATGTTGGGTTAAGTCCCGCAACGAGCGCAACCCTTGATTTTAGTTGCCAGCACTTAAAGGTGGGCACTCTAGAATGACTGCCGGTGACAAACCGGAGGAAGGCGGGGATGACGTCAAATCATCATGCCCCTTATGACCTGGGCTACACACGTACTACAATGGCCGGTACAACGGGCTGCGAAGGAGCGATCCGGAGCGAATCCTTAAAAGCCGGTCTCAGTTCGGATTGCAGGCTGCAACTCGCCTGCATGAAGTCGGAATTGCTAGTAATCGCGGATCAGCATGCCGCGGTGAATACGTTCCCGGGTCTTGTACACACCGCCCGTCACACCACGAGAGTTTACAACACCCGAAGCCGGTGGGGTAACCGCAAGGAGCCAGCCGTCGAAGGTGGGGTAGATGATTGGGGTGAAGTCGTAACAAGGTAGCCGTATCGGAAGG

>Paenibacillus radicis 694 (NR_148658.1)

GCTATACATGCAAGTCGAGCGGACTTGATGGAGTGCTTGCACTCCTGATGGTTAGCGGCGGACGGGTGAGTAACACGTGGGTAACCTGCCCATAAGACTGGGATAACATTCGGAAACGAATGCTAATACCGGATACGCGGTTTGGTCGCATGGCCGAATCGGGAAAGACGGAGCAATCTGTCACTTATGGATGGACCCGCGGCGCATTAGCTAGTTGGTGGGGTAACGGCTCACCAAGGCGACGATGCGTAGCCGACCTGAGAGGGTGATCGGCCACACTGGGACTGAGACACGGCCCAGACTCCTACGGGAGGCAGCAGTAGGGAATCTTCCGCAATGGACGAAAGTCTGACGGAGCAACGCCGCGTGAGTGATGAAGGTTTTCGGATCGTAAAGCTCTGTTGCCAGGGAAGAACGCTATGGAGAGTAACTGCTCCATAGGTGACGGTACCTGAGAAGAAAGCCCCGGCTAACTACGTGCCAGCAGCCGCGGTAATACGTAGGGGGCAAGCGTTGTCCGGAATTATTGGGCGTAAAGCGCGCGCAGGCGGCCTTGTAAGTCTGTCGTTTAAACTCGGAGCTCAACTCCGAGTCGCGATGGAAACTGCAAAGCTTGAGTGCAGAAGAGGAAAGTGGAATTCCACGTGTAGCGGTGAAATGCGTAGAGATGTGGAGGAACACCAGTGGCGAAGGCGACTTTCTGGGCTGTAACTGACGCTGAGGCGCGAAAGCGTGGGGAGCAAACAGGATTAGATACCCTGGTAGTCCACGCCGTAAACGATGAATGCTAGGTGTTAGGGGTTTCGATACCCTTGGTGCCGAAGTTAACACATTAAGCATTCCGCCTGGGGAGTACGGTCGCAAGACTGAAACTCAAAGGAATTGACGGGGACCCGCACAAGCAGTGGAGTATGTGGTTTAATTCGAAGCAACGCGAAGAACCTTACCAGGTCTTGACATCCCTTTGACCGTCCTAGAGATAGGGCTTTCCTTCGGGACAGAGGAGACAGGTGGTGCATGGTTGTCGTCAGCTCGTGTCGTGAGATGTTGGGTTAAGTCCCGCAACGAGCGCAACCCTTGATCTTAGTTGCCAGCACTTCGGGTGGGCACTCTAGGATGACTGCCGGTGACAAACCGGAGGAAGGTGGGGATGACGTCAAATCATCATGCCCCTTATGACCTGGGCTACACACGTACTACAATGGCCGATACAACGGGAAGCGAAACCGCGAGGTGGAGCCAATCCTATCAAAGTCGGTCTCAGTTCGGATTGCAGGCTGCAACTCGCCTGCATGAAGTCGGAATTGCTAGTAATCGCGGATCAGCATGCCGCGGTGAATACGTTCCCGGGTCTTGTACACACCGCCCGTCACACCACGAGAGTTTACAACACCCGAAGCCGGTGGGGTAACCGCAAGGAGCCAGCCGTCGAAG

>Paenibacillus lacus Agd-32 (NR_156905.1)

CATGCAAGTCGAGCGGACTTGATGGAGTGCTTGCACTCCTGATGGTTAGCGGCGGACGGGTGAGTAACACGTAGGCAACCTGCCCGACAGACCGGGATAACATTCGGAAACGAATGCTAATACCGGATAGGCGATCCTCTCGCATGAGGGGATCGAGAAAGACGGAGCAATCTGTCACTGTCGGATGGGCCTGCGGCGCATTAGCTAGTTGGTGGGGTAACGGCTCACCAAGGCGACGATGCGTAGCCGACCTGAGAGGGTGATCGGCCACACTGGGACTGAGACACGGCCCAGACTCCTACGGGAGGCAGCAGTAGGGAATCTTCCGCAATGGACGCAAGTCTGACGGAGCAACGCCGCGTGAGTGAGGAAGGCCTTCGGGTCGTAAAGCTCTGTTGCCAGGGAAGAACGGGTGGGAGAGTAACTGCTCCCGCCATGACGGTACCTGAGAAGAAAGCCCCGGCTAACTACGTGCCAGCAGCCGCGGTAATACGTAGGGGGCAAGCGTTGTCCGGAATTATTGGGCGTAAAGCGCGCGCAGGCGGCTTTGTAAGTCTTGTGTTTAAGTTCGGGGCTTAACCCCGTATCGCATGGGAAACTGCAAGGCTTGAGTGCAGAAGAGGAAAGTGGAATTCCACGTGTAGCGGTGAAATGCGTAGAGATGTGGAGGAACACCAGTGGCGAAGGCGACTTTCTGGGCTGTAACTGACGCTGAGGCGCGAAAGCGTGGGGAGCAAACAGGATTAGATACCCTGGTAGTCCACGCCGTAAACGATGAATGCTAGGTGTTAGGGGTTTCGATACCCTTGGTGCCGAAGTTAACACATTAAGCATTCCGCCTGGGGAGTACGGTCGCAAGACTGAAACTCAAAGGAATTGACGGGGACCCGCACAAGCAGTGGAGTATGTGGTTTAATTCGAAGCAACGCGAAGAACCTTACCAGGTCTTGACATCCCTCTGACCGGTCCAGAGATGGTCCTTCCCTTCGGGGCAGAGGAGACAGGTGGTGCATGGTTGTCGTCAGCTCGTGTCGTGAGATGTTGGGTTAAGTCCCGCAACGAGCGCAACCCTTGATCTTAGTTGCCAGCACTTCGGGTGGGCACTCTAAGATGACTGCCGGTGACAAACCGGAGGAAGGTGGGGATGACGTCAAATCATCATGCCCCTTATGACCTGGGCTACACACGTACTACAATGGCCGGTACAACGGGCTGCGAAATCGCGAGATGGAGCCAATCCCACCAAAGCCGGTCTCAGTTCGGATTGCAGGCTGCAACTCGCCTGCATGAAGTCGGAATTGCTAGTAATCGCGGATCAGCATGCCGCGGTGAATACGTTCCCGGGTCTTGTACACACCGCCCGTCACACCACGAGAGTTTACAACACCCGAAGTCGGTGGGGTAACCCGCAAGGGAGCCAGCCGCCGAAGGTGGGGTAGATGATTGGGGTGAAG

>Paenibacillus gorillae G1 (NR_136879.1)

AGAGTTTGATCCTGGCTCAGGACGAACGCTGGCGGCGTGCCTAATACATGCAAGTCGAGCGGACTTGATGAAGTGCTTGCACTCCTGATGGTTAGCGGCGGACGGGTGAGTAACACGTGGGTAACCTGCCCATAAGACTGGGATAACATTCGGAAACGAATGCTAATACCGGATACGCGGTTTGGTCGCATGGCCGAACCGGGAAAGACGGAGCAATCTGTCACTTATGGATGGACCCGCGGCGCATTAGCTAGTTGGTGGGGTAACGGCTCACCAAGGCGACGATGCGTAGCCGACCTGAGAGGGTGATCGGCCACACTGGGACTGAGACACGGCCCAGACTCCTACGGGAGGCAGCAGTAGGGAATCTTCCGCAATGGACGAAAGTCTGACGGAGCAACGCCGCGTGAGTGATGAAGGTTTTCGGATCGTAAAGCTCTGTTGCCAGGGAAGAACGCTATGGAGAGTAACTGCTCCATAGGTGACGGTACCTGAGAAGAAAGCCCCGGCTAACTACGTGCCAGCAGCCGCGGTAATACGTAGGGGGCAAGCGTTGTCCGGAATTATTGGGCGTAAAGCGCGCGCAGGCGGCCTTGTAAGTCTGTCGTTTAAACTCGGAGCTCAACTTCGAGTCGCGATGGAAACTGCAAAGCTTGAGTGCAGAAGAGGAAAGTGGAATTCCACGTGTAGCGGTGAAATGCGTAGAGATGTGGAGGAACACCAGTGGCGAAGGCGACTTTCTGGGCTGTAACTGACGCTGAGGCGCGAAAGCGTGGGGAGCAAACAGGATTAGATACCCTGGTAGTCCACGCCGTAAACGATGAATGCTAGGTGTTAGGGGTTTCGATACCCTTGGTGCCGAAGTTAACACATTAAGCATTCCGCCTGGGGAGTACGGTCGCAAGACTGAAACTCAAAGGAATTGACGGGGACCCGCACAAGCAGTGGAGTATGTGGTTTAATTCGAAGCAACGCGAAGAACCTTACCAGGTCTTGACATCCCTTTGACCGTCCTAGAGATAGGGCTTTCCTTCGGGACAAAGGAGACAGGTGGTGCATGGTTGTCGTCAGCTCGTGTCGTGAGATGTTGGGTTAAGTCCCGCAACGAGCGCAACCCTTGATCTTAGTTGCCAGCACTTCGGGTGGGCACTCTAGGATGACTGCCGGTGACAAACCGGAGGAAGGTGGGGATGACGTCAAATCATCATGCCCCTTATGACCTGGGCTACACACGTACTACAATGGCCGATACAACGGGAAGCGAAACCGCGAGGTGGAGCCAATCCTATCAAAGTCGGTCTCAGTTCGGATTGCAGGCTGCAACTCGCCTGCATGAAGTCGGAATTGCTAGTAATCGCGGATCAGCATGCCGCGGTGAATACGTTCCCGGGTCTTGTACACACCGCCCGTCACACCACGAGAGTTTACAACACCCGAAGCCGGTGGGGTAACCGCAAGGAGCCAGCCGTCGAAGGTGGGGTAGATGATTGGGGTGAAGTCGTAACAAGGTAGCCGT

>Paenibacillus translucens CJ11 (NR_159909.1)

TTGATGGAGTGCTTGCACTCCTGATGGTTAGCGGCGGACGGGTGAGTAACACGTAGGCAACCTGCCCGAC AGACCGGGATAACATTCGGAAACGAATGCTAATACCGGATAGGCGATAYCCTCGCATGAGGGAATCGAGA AAGACGGCGCAAGCTGTCACTGTCGGATGGGCCTGCGGCGCATTAGCTAGTTGGTGAGGTAACGGCTCAC CAAGGCGACGATGCGTAGCCGACCTGAGAGGGTGATCGGCCACACTGGGACTGAGACACGGCCCAGACTC CTACGGGAGGCAGCAGTAGGGAATCTTCCGCAATGGACGCAAGTCTGACGGAGCAACGCCGCGTGAGTGA GGAAGGCCTTCGGGTCGTAAAGCTCTGTTGCCAGGGAAGAACGGGTGGGAGAGTAACTGCTCCCGCCATG ACGGTACCTGAGAAGAAAGCCCCGGCTAACTACGTGCCAGCAGCCGCGGTAATACGTAGGGGGCAAGCGT TGTCCGGAATTATTGGGCGTAAAGCGCGCGCAGGCGGCTTTGTAAGTCTTGTGTTTAAGTTCGGGGCTTA ACCCCGTATCGCATGGGAAACTGCAAGGCTTGAGTGCAGAAGAGGAAAGTGGAATTCCACGTGTAGCGGT GAAATGCGTAGAGATGTGGAGGAACACCAGTGGCGAAGGCGACTTTCTGGGCTGTAACTGACGCTGAGGC GCGAAAGCGTGGGGAGCAAACAGGATTAGATACCCTGGTAGTCCACGCCGTAAACGATGAATGCTAGGTG TTAGGGGTTTCGATACCCTTGGTGCCGAAGTTAACACATTAAGCATTCCGCCTGGGGAGTACGGTCGCAA GACTGAAACTCAAAGGAATTGACGGGGACCCGCACAAGCAGTGGAGTATGTGGTTTAATTCGAAGCAACG CGAAGAACCTTACCAGGTCTTGACATCCCTCTGACCGGTCTAGAGATAGGCCTTCCCTTCGGGGCAGAGG AGACAGGTGGTGCATGGTTGTCGTCAGCTCGTGTCGTGAGATGTTGGGTTAAGTCCCGCAACGAGCGCAA CCCTTGATCTTAGTTGCCAGCACTTCGGGTGGGCACTCTAAGATGACTGCCGGTGACAAACCGGAGGAAG GTGGGGATGACGTCAAATCATCATGCCCCTTATGACCTGGGCTACACACGTACTACAATGGCCGGTACAA CGGGCTGCGAAATCGCGAGATGGAGCCAATCCCACCAAAGCCGGTCTCAGTTCGGATTGCAGGCTGCAAC TCGCCTGCATGAAGTCGGAATTGCTAGTAATCGCGGATCAGCATGCCGCGGTGAATACGTTCCCGGGTCT TGTACACACCGCCCGTCACACCACGAGAGTTTACAACACCCGAAGTCGGTGGGGTAACCCGCAAGGGAGC CAGCCGCCGAAG

>Paenibacillus oenotherae DT7-4 (NR_136822.1)

GCTCAGGACGAACGCTGGCGGCGTGCCTAATACATGCAAGTCGAGCGGACTTGACGAGAAGCTTGCTTCTCTGATGGTTAGCGGCGGACGGGTGAGTAACACGTAGGCAACCTGCCTGCAAGACCGGGATAACATTCGGAAACGAATGCTAATACCGGATACGCAATTTGGCTGCATGGCCGAATTGGGAAAGACGGAGCAATCTGTCACTTGCAGATGGGCCTGCGGCGCATTAGCTAGTTGGTGGGGTAACGGCTCACCAAGGCGACGATGCGTAGCCGACCTGAGAGGGTGATCGGCCACACTGGGACTGAGACACGGCCCAGACTCCTACGGGAGGCAGCAGTAGGGAATCTTCCGCAATGGACGAAAGTCTGACGGAGCAACGCCGCGTGAGTGATGAAGGTTCTCGGATCGTAAAGCTCTGTTGCCAGGGAAGAACACTTGAGAGAGTAACTGCTCTTGAGTTGACGGTACCTGAGAAGAAAGCCCCGGCTAACTACGTGCCAGCAGCCGCGGTAATACGTAGGGGGCAAGCGTTGTCCGGAATTATTGGGCGTAAAGCGCGCGCAGGCGGCTTTGTAAGTCTGGTGTTTAAGCTCGGGGCTCAACCCCGATTCGCACCGGAAACTGCAAGGCTTGAGTGCAGAAGAGGAAAGTGGAATTCCACGTGTAGCGGTGAAATGCGTAGAGATGTGGAGGAACACCAGTGGCGAAGGCGACTTTCTGGGCTGTAACTGACGCTGAGGCGCGAAAGCGTGGGGAGCAAACAGGATTAGATACCCTGGTAGTCCACGCCGTAAACGATGAATGCTAGGTGTTAGGGGTTTCAATACCCTTGGTGCCGAAGTTAACACATTAAGCATTCCGCCTGGGGAGTACGCTCGCAAGAGTGAAACTCAAAGGAATTGACGGGGACCCGCACAAGCAGTGGAGTATGTGGTTTAATTCGAAGCAACGCGAAGAACCTTACCAGGTCTTGACATCCCTCTGAATCCTCTAGAGATAGAGGCGGCCTTCGGGACAGAGGAGACAGGTGGTGCATGGTTGTCGTCAGCTCGTGTCGTGAGATGTTGGGTTAAGTCCCGCAACGAGCGCAACCCTTGATTTTAGTTGCCAGCACTTTGGGTGGGCACTCTAGAATGACTGCCGGTGACAAACCGGAGGAAGGCGGGGATGACGTCAAATCATCATGCCCCTTATGACCTGGGCTACACACGTACTACAATGGCCGGTACAACGGGCCGCGAAGCCGCGAGGTGGAGCTAATCCTAAAAAGCCGGTCTCAGTTCGGATTGCAGGCTGCAACTCGCCTGCATGAAGTCGGAATTGCTAGTAATCGCGGATCAGCATGCCGCGGTGAATACGTTCCCGGGTCTTGTACACACCGCCCGTCACACCACGAGAGTTTACAACACCCGAAGTCGGTGGGGTAACCGCAAGGAGCCAGCCGCCGAAGGTGGGGTAGATGATTGGGGTGAAGTC

>Paenibacillus thailandensis S3-4A (NR_041490.1)

CCTGGCTCAGGACGAACGCTGGCGGCGTGCCTAATACATGCAAGTCGAGCGGAGTTGATGGAGGTGCTTGCACTTCTGANGGTTAGCGGCGGACGGGTGAGTAACACGTAGGTAACCTGCCCATAAGACCGGGATAACATTCGGAAACGGATGCTAATACCGGATACGCAATTCTCTCGCATGAGGGGATTGGGAAAGGCGGAGCAATCTGTCACTTATGGATGGACCTGCGGCGCATTAGCTAGTTGGTGAGGTAACGGCTCACCAAGGCGACGATGCGTAGCCGACCTGAGAGGGTGATCGGCCACACTGGGACTGAGACACGGCCCAGACTCCTACGGGAGGCAGCAGTAGGGAATCTTCCGCAATGGGCGAAAGCCTGACGGAGCAACGCCGCGTGAGTGATGAAGGTTTTCGGATCGTAAAGCTCTGTTGCCAGGGAAGAACGCTTGGGAGAGTAACTGCTCTCAAGGTGACGGTACCTGAGAAGAAAGCCCCGGCTAACTACGTGCCAGCAGCCGCGGTAATACGTAGGGGGCAAGCGTTGTCCGGAATTATTGGGCGTAAAGCGCGCGCAGGCGGTTCATTAAGTCTGGTGTTTAAGGCTGGGGCTCAACCCCGGTTCGCACTGGAAACTGGTGGACTTGAGTGCAGAAGAGGAAAGTGGAATTCCACGTGTAGCGGTGAAATGCGTAGAGATGTGGAGGAACACCAGTGGCGAAGGCGACTTTCTGGGCTGTAACTGACGCTGAGGCGCGAAAGCGTGGGGAGCAAACAGGATTAGATACCCTGGTAGTCCACGCCGTAAACGATGATTGCTAGGTGTTAGGGGTTTCGATACCCTTGGTGCCGAAGTTAACACATTAAGCATTCCGCCTGGGGAGTACGGTCGCAAGACTGAAACTCAAAGGAATTGACGGGGACCCGCACAAGCAGTGGAGTATGTGGTTTAATTCGAAGCAACGCGAAGAACCTTACCAGGTCTTGACATCCCCCTGACCGGTCTAGAGATAGGCCTTTCCTTCGGGACAGGGGAGACAGGTGGTGCATGGTTGTCGTCAGCTCGTGTCGTGAGATGTTGGGTTAAGTCCCGCAACGAGCGCAACCCTTGATCTTAGTTGCCAGCACTTTGGGTGGGCACTCTAGGATGACTGCCGGTGACAAACCGGAGGAAGGTGGGGATGACGTCAAATCATCATGCCCCTTATGACCTGGGCTACACACGTACTACAATGGCCGGTACAACGGGAAGCGAAGGAGCGATCCGGAGCCAATCCTATAAAGCCGGTCTCAGTTCGGATTGCAGGCTGCAACTCGCCTGCATGAAGTCGGAATTGCTAGTAATCGCGGATCAGCATGCCGCGGTGAATACGTTCCCGGGTCTTGTACACACCGCCCGTCACACCACGAGAGTTTACAACACCCGAAGCCGGTGGGGTAACCGCAAGGAGCCAGCCGTCGAAGGTGGGGTAGATGATTGGGGTG

>Paenibacillus castaneae Ch-32 (NR_044403.1)

TAGAGTTTGATCCTGGCTCAGGACGAACGCTGGCGGCGTGCCTAATACATGCAAGTCGAGCGGAGTTGAAGAGGTGCTTGCACCTCTGATACTTAGCGGCGGACGGGTGAGTAACACGTGGGTAACCTGCCTTTAAGACTGGGATAACATTCGGAAACGAATGCTAATACCGGATACGCGATACGGTCGCATGACTGAATCGGGAAAGATGGAGCAATCTATCACTTAGAGATGGACCCGCGGCGCATTAGCTAGTTGGTGAGGTAACGGCTCACCAAGGCGACGATGCGTAGCCGACCTGAGAGGGTGATCGGCCACACTGGGACTGAGACACGGCCCAGACTCCTACGGGAGGCAGCAGTAGGGAATCTTCCGCAATGGACGAAAGTCTGACGGAGCAACGCCGCGTGAGTGATGAAGGTTTTCGGATCGTAAAGCTCTGTTGCCAGGGAAGAATGCTTGGGAGAGTAACTGCTCTCAAGGTGACGGTACCTGAGAAGAAAGCCCCGGCTAACTACGTGCCAGCAGCCGCGGTAATACGTAGGGGGCAAGCGTTGTCCGGAATTATTGGGCGTAAAGCGCGCGCAGGCGGCCTTGTAAGTCTGTTGTTTAAACTCGGGGCTCAACCCCGAGTCGCAATGGAAACTGCAAAGCTTGAGTACAGAAGAGGAAAGTGGAATTCCACGTGTAGCGGTGAAATGCGTAGAGATGTGGAGGAACACCAGTGGCGAAGGCGACTTTCTGGGCTGTAACTGACGCTGAGGCGCGAAAGCGTGGGGAGCAAACAGGATTAGATACCCTGGTAGTCCACGCCGTAAACGATGAATGCTAGGTGTTAGGGGTTTCAATACCCTTGGTGCCGAAGTTAACACATTAAGCATTCCGCCTGGGGAGTACGGTCGCAAGACTGAAACTCAAAGGAATTGACGGGGACCCGCACAAGCAGTGGAGTATGTGGTTTAATTCGAAGCAACGCGAAGAACCTTACCAGGTCTTGACATCCCTCTGACCGTCCTAGAGATAGGGCTTTCCTTCGGGACAGAGGAGACAGGTGGTGCATGGTTGTCGTCAGCTCGTGTCGTGAGATGTTGGGTTAAGTCCCGCAACGAGCGCAACCCTTGATCTTAGTTGCCAGCACTTCGGGTGGGCACTCTAGGATGACTGCCGGTGACAAACCGGAGGAAGGTGGGGATGACGTCAAATCATCATGCCCCTTATGACCTGGGCTACACACGTACTACAATGGCCGATACAACGGGAAGCGAAACCGCGAGGTGGAGCCAATCCTATCAAAGTCGGTCTCAGTTCGGATTGCAGGCTGCAACTCGCCTGCATGAAGTCGGAATTGCTAGTAATCGCGGATCAGCATGCCGCGGTGAATACGTTCCCGGGTCTTGTACACACCGCCCGTCACACCACGAGAGTTTACAACACCCGAAGCCGGTGGGGTAACCCGCAAGGGAGCTAGCCGTCGAAGGTGGGGTAGATGATTGGGGTGAAGTCGTAACAAGGTAGCCGTATCGGAAGGTGCGGCTGGATCACCTCCTTA

>Paenibacillus xanthinilyticus 11N27 (NR_137226.1)

CAGGACGAACGCTGGCGGCGTGCCTAATACATGCAAGTCGAGCGGACTTGAGGAGAAGCTTGCTTCTCCGATGGTTAGCGGCGGACGGGTGAGTAACACGTAGGCAACCTGCCTGCAAGACCGGGATAACATTCGGAAACGAATGCTAATACCGGATACGCAATTCGGCCGCATGGCTGGATTGGGAAAGACGGAGCAATCTGTCACTTGCAGATGGGCCTGCGGCGCATTAGCTAGTTGGTGGGGTAACGGCTCACCAAGGCGACGATGCGTAGCCGACCTGAGAGGGTGATCGGCCACACTGGGACTGAGACACGGCCCAGACTCCTACGGGAGGCAGCAGTAGGGAATCTTCCGCAATGGACGAAAGTCTGACGGAGCAACGCCGCGTGAGTGATGAAGGTTCTCGGATCGTAAAGCTCTGTTGCCAGGGAAGAACACTTGGAAGAGTAACTGCTTCTGAGTTGACGGTACCTGAGAAGAAAGCCCCGGCTAACTACGTGCCAGCAGCCGCGGTAATACGTAGGGGGCAAGCGTTGTCCGGAATTATTGGGCGTAAAGCGCGCGCAGGCGGCTTTGTAAGTCTGGTGTTTAAGCTCGGGGCTCAACCCCGATTCGCACCGGAAACTGCAAGGCTTGAGTGCAGAAGAGGAAAGTGGAATTCCACGTGTAGCGGTGAAATGCGTAGAGATGTGGAGGAACACCAGTGGCGAAGGCGACTTTCTGGGCTGTAACTGACGCTGAGGCGCGAAAGCGTGGGGAGCAAACAGGATTAGATACCCTGGTAGTCCACGCCGTAAACGATGAATGCTAGGTGTTAGGGGTTTCAATACCCTTGGTGCCGAAGTTAACACATTAAGCATTCCGCCTGGGGAGTACGCTCGCAAGAGTGAAACTCAAAGGAATTGACGGGGACCCGCACAAGCAGTGGAGTATGTGGTTTAATTCGAAGCAACGCGAAGAACCTTACCAGGTCTTGACATCCCTCTGACCGGTCTAGAGATAGGCCTTTCCTTCGGGACAGAGGAGACAGGTGGTGCATGGTTGTCGTCAGCTCGTGTCGTGAGATGTTGGGTTAAGTCCCGCAACGAGCGCAACCCTTGATTTTAGTTGCCAGCACGTAAGGGTGGGCACTCTAAAATGACTGCCGGTGACAAACCGGAGGAAGGCGGGGATGACGTCAAATCATCATGCCCCTTATGACCTGGGCTACACACGTACTACAATGGCCGGTACAACGGGCTGCGAACCCGCGAGGTGGAGCTAATCCTAAAAAGCCGGTCTCAGTTCGGATTGCAGGCTGCAACTCGCCTGCATGAAGTCGGAATTGCTAGTAATCGCGGATCACCATGCCGCGGTGAATACTTTCCCGGGTCTTGTACACACCGCCCGTCACACCACGAGAGTTTACAACACCCGAAGTCGGTGGGGTAACCGCAAGGAGCCAGCCGCCAAAGGTGGGGTAGATGATTGGGGTGAA

>Paenibacillus xinjiangensis B538 (NR_043221.1)

TTAGAGTTTGATCCCCTGCTCAGGACGAACGCTGGCGGCGTGCCTAATACATGCAAGTCGAGCGGACTTGATGGAGTGCTTGCACTCCTGATAGTTAGCGGCGGACGGGTGAGTAACACGTGGGTAACCTGCCCATAAGACTGGGATAACATTCGGAAACGAATGCTAATACCGGATACGCAATTTGGTCGCATGGCCGARTTGGGAAAGGCGGAGCAATCTGCCACTTATGGATGGACCTGCGGTGCATTAGCTAGTTGGTGGGGTAACGGCTCACCAAGGCGACGATGCATAGCCGACCTGAGAGGGTGATCGGCCACACTGGGACTGAGACACGGCCCAGACTCCTACGGGAGGCAGCAGTAGGGAATCTTCCGCAATGGACGAAAGTCTGACGGAGCAACGCCGCGTGAGTGATGAAGGTTTTCGGATCGTAAAGCTCTGTTGCCAGGGAAGAACGCTATGGAGAGTAACTGCTCCATAGGTGACGGTACCTGAGAAGAAAGCCCCGGCTAACTACGTGCCAGCAGCCGCGGTAATACGTAGGGGGCAAGCGTTGTCCGGAATTATTGGGCGTAAAGCGCGCGCAGGCGGCCTTGTAAGTCTGTTGTTTAAACTCGGAGCTCAACTTCGAGTCGCAATGGAAACTGCAAAGCTTGAGTACAGAAGAGGAAAGTGGAATTCCACGTGTAGCGGTGAAATGCGTAGAGATGTGGAGGAACACCAGTGGCGAAGGCGACTTTCTGGGCTGTAACTGACGCTGAGGCGCGAAAGCGTGGGGAGCAAACAGGATTAGATACCCTGGTAGTCCACGCCGTAAACGATGAATGCTAGGTGTTAGGGGTTTCGATACCCTTGGTGCCGAAGTTAACACATTAAGCATTCCGCCTGGGGAGTACGGTCGCAAGACTGAAACTCAAAGGAATTGACGGGGACCCGCACAAGCAGTGGAGTATGTGGTTTAATTCGAAGCAACGCGAAGAACCTTACCAGGTCTTGACATCCCTCTGACCGTCCTAGAGATAGGACTTTCCTTCGGGACAGAGGAGACAGGTGGTGCATGGTTGTCGTCAGCTCGTGTCGTGAGATGTTGGGTTAAGTCCCGCAACGAGCGCAACCCTTGATCTTAGTTGCCAGCACTTTGGGTGGGCACTCTAGGATGACTGCCGGTGACAAACCGGAGGAAGGTGGGGATGACGTCAAATCATCATGCCCCTTATGACCTGGGCTACACACGTACTACAATGGCCGATACAACGGGTCGCGAAACCGCGAGGTGGAGCCAATCCCARCAAAGTCGGTCTCAGTTCGGATTGCAGGCTGCAACTCGCCTGCATGAAGTCGGAATTGCTAGTAATCGCGGATCAGCATGCCGCGGTGAATACGTTCCCGGGTCTTGTACACACCGCCCGTCACACCACGAGAGTTTACAACACCCGAAGCCGGTGGGGTAACCGCAAGGAGCCAGCCGTCGAAGGTGGGGTAGATGATTGGGGTGAAGTCGTAACAAGGTAACCAGGG

>Paenibacillus xanthanilyticus AS7 (NR_159286.1)

ATACATGCAAGTCGAGCGGACTACACCTTCGGGTGTGGTTAGCGGCGGACGGGTGAGTAACACGTAGGTAACCTGCCTGTAAGACTGGGATAACATTCGGAAACGAATGCTAATACCGGATATGCGGTTTGCTCGCATGAGCGAATCGGGAAAGACGGTGCAAGCTGTCACTTACAGATGGACCTGCGGCGCATTAGCTAGTTGGTGGGGTAACGGCTCACCAAGGCGACGATGCGTAGCCGACCTGAGAGGGTGATCGGCCACACTGGGACTGAGACACGGCCCAGACTCCTACGGGAGGCAGCAGTAGGGAATCTTCCGCAATGGACGAAAGTCTGACGGAGCAACGCCGCGTGAGTGATGAAGGTTTTCGGATCGTAAAGCTCTGTTGCCAGGGAAGAACGAGTGGGAGAGTAACTGCTCCTGCTATGACGGTACCTGAGAAGAAAGCCCCGGCTAACTACGTGCCAGCAGCCGCGGTAATACGTAGGGGGCAAGCGTTGTCCGGAATTATTGGGCGTAAAGCGCGCGCAGGCGGCTTTGTAAGTCAGGTGTTTAATCTCGGGGCTCAACCCCGATTCGCATCTGAAACTGCAAGGCTTGAGTGCAGAAGAGGAAAGTGGAATTCCACGTGTAGCGGTGAAATGCGTAGAGATGTGGAGGAACACCAGTGGCGAAGGCGACTTTCTGGGCTGTAACTGACGCTGAGGCGCGAAAGCGTGGGGAGCAAACAGGATTAGATACCCTGGTAGTCCACGCCGTAAACGATGAATGCTAGGTGTTAGGGGTTTCGATACCCTTGGTGCCGAAGTTAACACATTAAGCATTCCGCCTGGGGAGTACGCTCGCAAGAGTGAAACTCAAAGGAATTGACGGGGACCCGCACAAGCAGTGGAGTATGTGGTTTAATTCGAAGCAACGCGAAGAACCTTACCAGGTCTTGACATCCCTCTGAATCACCTAGAGATAGGGGCGGCCCTTCGGGGACAGAGGAGACAGGTGGTGCATGGTTGTCGTCAGCTCGTGTCGTGAGATGTTGGGTTAAGTCCCGCAACGAGCGCAACCCTTGATTTTAGTTGCCAGCACTTCGGGTGGGCACTCTAGAATGACTGCCGGTGACAAACCGGAGGAAGGCGGGGATGACGTCAAATCATCATGCCCCTTATGACCTGGGCTACACACGTACTACAATGGCCGTTACAACGGGAAGCGAAGCCGCGAGGTGGAGCGAATCCTAAAAAGGCGGTCTCAGTTCGGATTGCAGGCTGCAACTCGCCTGCATGAAGTCGGAATTGCTAGTAATCGCGGATCAGCATGCCGCGGTGAATACGTTCCCGGGTCTTGTACACACCGCCCGTCACACCACGAGAGTTTACAACACCCGAAGCCGGTGGGGTAACCGCAAGGAGCCAGCCGTCGAAGGTGGC

>Paenibacillus tarimensis SA-7-6 (NR_044102.1)

GCAAGTCGAGCGGACTTGAAGAGGAGCTTGCTCCTCTGATAGTTAGCGGCGGACGGGTGAGTAACACGTAGGCAACCTGCCTGTAAGACTGGGATAACATCCGGAAACGGATGCTAATACCGGATACGCGGTTCCCCCGCATGAGGGAACCGGGAAAGACGGCGTAAGCTGTCACTTGCAGATGGGCCTGCGGCGCATTAGCTAGTTGGTGGGGTAACGGCCTACCAAGGCGACGATGCGTAGCCGACCTGAGAGGGTGATCGGCCACACTGGGACTGAGACACGGCCCAGACTCCTACGGGAGGCAGCAGTAGGGAATCTTCCGCAATGGACGAAAGTCTGACGGAGCAACGCCGCGTGAGTGAGGAAGGCCTTCGGGTCGTAAAGCTCTGTTGCCAGGGAAGAACGGGTGGAGGAGTAACTGCCTCCGCCATGACGGTACCTGAGAAGAAAGCCCCGGCTAACTACGTGCCAGCAGCCGCGGTAATACGTAGGGGGCAAGCGTTGTCCGGAATTATTGGGCGTAAAGCGCGCGCAGGCGGCTTTGTAAGTCTTGTGTTTAAACTCGGGGCTCAACCTCGAGTCGCATGGGAAACTGCAGGGCTTGAGTGCAGAAGAGGAAAGTGGAATTCCACGTGTAGCGGTGAAATGCGTAGAGATGTGGAGGAACACCAGTGGCGAAGGCGACTTTCTGGGCTGTAACTGACGCTGAGGCGCGAAAGCGTGGGGAGCAAACAGGATTAGATACCCTGGTAGTCCACGCCGTAAACGATGAATGCTAGGTGTTAGGGGTTTCGATACCCTTGGTGCCGAAGTTAACACATTAAGCATTCCGCCTGGGGAGTACGGTCGCAAGACTGAAACTCAAAGGAATTGACGGGGACCCGCACAAGCAGTGGAGTATGTGGTTTAATTCGAAGCAACGCGAAGAACCTTACCAGGTCTTGACATCCCTCTGACCGGTCCAGAGATAGGCCTTTCCTTCGGGACAGAGGAGACAGGTGGTGCATGGTTGTCGTCAGCTCGTGTCGTGAGATGTTGGGTTAAGTCCCGCAACGAGCGCAACCCTTGATCTTAGTTGCCAGCAYTTCGGGTGGGCACTCTAAGATGACTGCCGGTGACAAACCGGAGGAAGGTGGGGATGACGTCAAATCATCATGCCCCTTATGACCTGGGCTACACACGTACTACAATGGCCGATACAACGGGTCGCGAAGCCGCGAGGTGGAGCCAATCCTATCAAAGTCGGTCTCAGTTCGGATTGCAGGCTGCAACTCGCCTGCATGAAGTCGGAATTGCTAGTAATCGCGGATCAGCATGCCGCGGTGAATACGTTCCCGGGTCTTGTACACACCGCCCGTCACACCACGAGAGTTTACAACACCCGAAGCCGGTGGGGTAACCGCAAGGAGCCAGCCGTCGAAGGTGGGGTAGATGATTGGGGTGAAGTCGTAAC

>Escherichia coli K-12 (LT899983.1)

GTCGAACGGTAACAGGAAGCAAGCTTGCTNCTTNGCTGACGAGTGGCGGACGGGTGAGTAATGTCTGGGAAACTGCCTGATGGAGGGGGATAACTACTGGAAACGGTAGCTAATACCGCATAACGTCGCAAGACCAAAGAGGGGGACCTTCGGGCCTCTTGCCATCGGATGTGCCCAGATGGGATTAGCTNGTNGGTGGGGTAACGGCTCACCTAGGCGACGATCCCTAGCTGGTCTGAGAGGATGACCAGCCACACTGGAACTGAGACACGGTCCAGACTCCTACGGGAGGCAGCAGTGGGGAATATTGCACAATGGGCGCAAGCCTGATGCAGCCATGCCGCGTGTATGAAGAAGGCCTTCGGGTTGTAAAGTACTTTCAGCGGGGAGGAAGGGAGTAAAGTTAATACCTTTGCTCATTGACGTTACCCGCAGAAGAAGCACCGGCTAACTCCGTGCCAGCAGCCGCGGTAATACGGAGGGTGCAAGCGTTAATCGGAATTACTGGGCGTAAAGCGCACGCAGGCGGTTTGTTAAGTCAGATGTGAAATCCCCGGGCTCAACCTGGGAACTGCATCTGATACTGGCAAGCTTGAGTCTCGTAGAGGGGGGTAGAATTCCAGGTGTAGCGGTGAAATGCGTAGAGATCTGGAGGAATACCGGTGGCGAAGGCGGCCCCCTGGACGAAGACTGACGCTCAGGTGCGAAAGCGTGGGGAGCAAACAGGATTAGATACCCTGGTAGTCCACGCCGTAAACGATGTCGACTTGGAGGTTGTGCCCTTGAGGCGTGGCTTCCGGAGCTAACGCGTTAAGTCGACCGCCTGGGGAGTACGGCCGCAAGGTTAAAACTCAAATGAATTGACGGGGGCCCGCACAAGCGGTGGAGCATGTGGTTTAATTCGATGCAACGCGAAGAACCTTACCTGGTCTTGACATCCACGGAAGTTTTCAGAGATGAGAATGTGCCTTCGGGAACCGTGAGACAGGTGCTGCATGGCTGTCGTCAGCTCGTGTTGTGAAATGTTGGGTTAAGTCCCGCAACGAGCGCAACCCTTATCCTTTGTTGCCAGCGGTCCGGCCGGGAACTCAAAGGAGACTGCCAGTGATAAACTGGAGGAAGGTGGGGATGACGTCAAGTCATCATGGCCCTTACGACCAGGGCTACACACGTGCTACAATGGCGCATACAAAGAGAAGCGACCTCGCGAGAGCAAGCGGACCTCATAAAGTGCGTCGTAGTCCGGATTGGAGTCTGCAACTCGACTCCATGAAGTCGGAATCGCTAGTAATCGTGGATCAGAATGCCACGGTGAATACGTTCCCGGGCCTTGTACACACCGCCCGTCACACCATGGGAGTGGGTTGCAAAAGAAGTAGGTAGCTTAACCTTCGGGAGGGCGCTTACCACTTTGTGATTCATGACTGGGGT
